# Comparative Analysis of Two CO_2_ Sequestration Pathways for Magnesium Slag Based on Kinetics and Life Cycle Assessment

**DOI:** 10.3390/ma19010193

**Published:** 2026-01-05

**Authors:** Zhen Lu, Yan Wu, Hongshuo Ding, Chengyuan Zhao, Yunlong Bai, Li Zhang

**Affiliations:** 1 School of Metallurgy, Northeastern University, Shenyang 110819, China; 2 Key Laboratory for Ecological Metallurgy of Multimetallic Mineral, Ministry of Education, Northeastern University, Shenyang 110819, China

**Keywords:** magnesium slag, carbon dioxide sequestration, kinetics, life cycle assessment (LCA), dual carbonation pathways

## Abstract

As a metallurgical solid waste rich in active calcium oxide, magnesium slag (MS) is endowed with significant carbon dioxide sequestration potential due to its inherent properties, providing a feasible path for the simultaneous solution of waste residue disposal and carbon dioxide emission reduction. However, current research has neither clarified the kinetic mechanism (core theoretical support for carbon dioxide sequestration industrialization) nor systematically evaluated the life cycle environmental impacts of MS’s two carbonation routes (direct or indirect leaching carbonation). To address this, this study explores kinetic laws via the single-factor control variable method, and combines life cycle assessment (LCA) to fill the gap, providing key theoretical support for process optimization and engineering promotion. Kinetic results show indirect carbon dioxide sequestration (ICDS) forms an inert silicon-rich layer (core-shrinkage model, mixed control, 28.4 kJ/mol activation energy), while direct carbon dioxide sequestration (DCDS) involves dual-layer formation and pore blockage (mixed control, 14.0 kJ/mol). The ICDS achieves a higher reaction rate of 89%, compared to 63% for the DCDS. In life cycle assessments, DCDS demonstrates outstanding overall environmental sustainability, particularly excelling in carbon dioxide sequestration and acidification control, while ICDS exhibits significant environmental drawbacks (such as high carbon dioxide emissions and ecological toxicity). However, ICDS possesses advantages such as high feedstock utilization and strong synthesis capabilities for high-value-added products. Through targeted optimization, its environmental indicators can be reduced in the future, making it suitable for specific scenarios like high-end calcium carbonate production and resource utilization of low-grade magnesium slag.

## 1. Introduction

As the third most widely used metal after steel and aluminum, magnesium, with its light weight, high strength, and excellent corrosion resistance, plays a crucial role in emerging industries such as new energy vehicles, aerospace, and electronic devices [1]. China dominates the global magnesium production, accounting for over 85% of the total output, among which the Pidgeon process is the main smelting technology [2]. However, the rapid development of the magnesium industry is accompanied by two pressing challenges: the massive generation of magnesium slag (MS) and the escalating pressure of carbon dioxide (CO_2_) emission reduction. Approximately 6–8 tons of MS are produced per ton of metallic magnesium [3]. The stockpiling of MS occupies a large amount of valuable land resources, and the slag tends to pulverize in the natural environment. Its fine particles are prone to being scattered by the wind, causing air pollution, affecting air quality, and endangering human respiratory health [4]. The massive emission and accumulation of MS have become a key bottleneck restricting the sustainable development of the magnesium industry.

As an industrial by-product, MS has emerged as an ideal material for CO_2_ sequestration due to its chemical similarity to steel slag and high reactivity with CO_2_ [5,6]. Alkaline in nature and rich in magnesium and calcium silicates, MS can form stable carbonates through wet carbonation, achieving the dual benefits of waste resource utilization and greenhouse gas emission reduction [7]. Recent studies have shown that MS can achieve a carbon dioxide sequestration capacity of up to 22.14% (mass fraction) through carbonation. The reaction products-mainly calcium carbonate (calcite and aragonite) and highly polymerized calcium silicate hydrate (C-S-H) gels-not only fix CO_2_ but also enhance the mechanical properties of the material [8]. The carbonation mechanism involves the dissolution of active MgO and CaO phases in MS, followed by carbonate precipitation that densifies the microstructure and improves compressive strength. This allows MS to replace up to 30% (mass fraction) of clinker in cement formulations without compromising performance [6].

From an environmental perspective, the MS carbonation process aligns with the concept of the circular economy by converting waste into value-added products. For example, ladle furnace slag (LFS)-another by-product of the steel industry-can absorb 8% (mass fraction) of CO_2_ and immobilize heavy metals such as lead (Pb) through carbonate encapsulation, reducing leaching concentrations by four orders of magnitude [9]. Similarly, MS-based materials for mine backfilling exhibit a CO_2_ sequestration rate of 14.55% (mass fraction), along with improved rheological properties and early strength development (reaching 8.854 MPa after 7 days of carbonation curing) [7]. These advancements highlight the scalability of MS carbonation, especially in industrial scenarios where waste resources and CO_2_ emissions intersect.

Despite significant progress in understanding the carbonation efficiency and product formation of MS through mineral carbonation [10], critical gaps remain in quantifying carbonation kinetics and evaluating the environmental footprint of the process. Existing studies have confirmed that mechanochemical activation and optimization of carbonation conditions can enhance the reactivity of calcium/magnesium silicates in MS, doubling CO_2_ absorption [11]. However, the temporal dynamic characteristics of the carbonation process (such as reaction rate and intermediate phase evolution) in both direct carbonation and indirect leaching carbonation pathways have not been systematically quantified. For instance, early exposure of the MgO-SiO_2_ system to CO_2_ accelerates the formation of hydrated magnesium hydroxycarbonate (HMHC) but reduces the content of M-S-H gels, indicating a trade-off between carbonation rate and material stability [12]. Yet, the differences in such laws between the two MS carbonation pathways lack in-depth analysis. In addition, although MS-based materials demonstrate excellent CO_2_ sequestration capacity and mechanical properties [7], the life cycle assessment (LCA) of their entire chain-including energy input, CO_2_ emissions from pretreatment (e.g., grinding), and long-term stability of carbonation products-remains insufficiently explored. Notably, innovative technologies such as thermochemical coupling have achieved an energy efficiency of 47.6% in CO_2_ mineralization [7,13,14,15], providing new ideas for reducing the energy consumption of MS carbonation processes. However, the large-scale integration of such technologies with direct/indirect MS carbonation still requires verification.

The existence of these aforementioned gaps has hindered the large-scale application of MS carbonation technology in fields such as construction and mining. Addressing the core issues of insufficient systematic quantification of the kinetic mechanisms of direct carbonation and indirect leaching carbonation, as well as the unclear full-chain environmental impacts in LCA, this study aims to achieve “high-efficiency CO_2_ sequestration of MS coupled with low-CO_2_ emission” and conducts two key research tasks: firstly, systematically investigate the kinetic characteristics of both direct carbonation and indirect leaching carbonation processes, clarifying the rate-determining steps of reaction rate. Establish kinetic models for the two carbonation pathways to quantify the correlation between CO_2_ sequestration efficiency and reaction rate under different conditions. Secondly, construct a full-LCA framework for the MS carbonation process, covering the entire stages of raw material pretreatment, carbonation reaction, and product application, to evaluate its energy consumption, carbon dioxide emissions, and environmental sustainability. This study innovatively integrates kinetic mechanism quantification and full-chain LCA for the first time, systematically clarifying the rate-determining steps of two carbonation pathways and their environmental footprint differences. Unlike previous studies that only focus on carbonation efficiency, this work realizes the dual breakthrough of ‘theoretical mechanism + environmental impact’ evaluation, providing a comprehensive decision-making basis for industrial application of MS carbon sequestration technology.

## 2. Materials and Methods

### 2.1. Experimental Raw Materials

The raw material utilized in this study is the MS generated during the production of metallic magnesium via the Pidgeon process at a non-ferrous metal smelter located in Yulin, Shaanxi Province, China. Raw MS was first ground in a ball mill for 5–8 min to produce a fine powder, then screened through a 200-mesh sieve to control particle size below 74 microns. The screened slag powder was thoroughly mixed and dried for subsequent analysis and experimentation. The chemical composition of the MS used in the experiments is shown in Table 1.

### 2.2. Experimental Method

Mineral carbonation achieves permanent CO_2_ sequestration by reacting CO_2_ with calcium- and magnesium-rich silicate minerals or industrial wastes (e.g., steel slag, fly ash) to form stable carbonates. Its technical routes are mainly classified into the indirect and direct methods.

#### 2.2.1. Indirect Method

The indirect CO_2_ sequestration (ICDS) first leaches calcium and magnesium ions from MS via chemical extraction (e.g., acid or alkali dissolution), then reacts these ions with CO_2_ in a separate reactor to precipitate as carbonates. The process consists of two steps: “dissolution and precipitation”. In the ICDS experiments of this study, ammonium chloride solution was used as the leaching agent, mixed and stirred with MS powder at a liquid-to-solid ratio of 4:1. For the calcium ion leaching stage, five groups of experimental temperatures were set: 30 °C, 50 °C, 70 °C, 90 °C, and 100 °C. The calcium ion precipitation experiments were uniformly conducted at room temperature, reacting for a certain time under an atmosphere of 99.9% pure CO_2_. Due to the short reaction time and high precipitation rate of calcium ions in calcium chloride solutions, this process is not a controlling step in indirect carbonation. Therefore, the kinetic analysis of indirect carbonation focuses solely on the leaching process. The leaching efficiency of calcium ions from the MS can be equated to the slag’s CO_2_ sequestration capacity. In this study, ethylenediaminetetraacetic acid (EDTA) titration was employed to analyze the calcium ion leaching efficiency of the two CO_2_ sequestration methods. The reaction rate of CaO in MS is denoted by *X* and calculated using Formula (1).

(1) $$X=\frac{Vw_{1}}{m_{0}w_{0}}\times 100\%$$

Among these, X: reaction rate of CaO in MS, %; *m*_0_: mass of MS, g; *w*_0_: calcium content in MS, %; *V*: volume of calcium leachate, L; *w*_1_: calcium content in calcium leachate, g/L.

#### 2.2.2. Direct Method

The direct CO_2_ sequestration (DCDS) involves directly introducing CO_2_ into a single reactor to induce carbonation reactions with minerals or wastes, which can be divided into two forms: gas–solid phase direct carbonation and aqueous phase direct carbonation. For the DCDS experiments, purified water was used as the leaching agent, mixed and stirred with MS powder at a liquid-to-solid ratio of 15:1. Three groups of experimental temperatures were set: 30 °C, 60 °C, and 90 °C, with reactions carried out for a certain time under an atmosphere of 99.9% pure CO_2_.

The reaction rate of calcium oxide in MS was determined by thermogravimetric analysis (TGA). To eliminate interference from inherent crystalline water in the mineral, samples directly after solid CO_2_ fixation were first heated to 500 °C and held at this temperature for 12 h to remove crystalline water. The samples were then weighed and their mass recorded. The samples were then heated to 1000 °C, held at this temperature for 12 h, and weighed again. Since the decomposition temperature range of CaCO_3_ primarily spans between 600 °C and 900 °C, mass loss within this temperature range during the latter stage was attributed to the decomposition of CaCO_3_ in the samples [16]. The amount of CO_2_ directly absorbed per 100 g of MG was calculated and converted to the reaction rate of CaO using Equation (2):

(2) $$X=\frac{m_{500}-m_{1000}}{m_{1000}}\times (\frac{56}{w_{0}\times 44})\times 100\%$$

X: reaction rate of CaO in MS, %; *w*_0_: calcium content in MS, %; *m*_500_ and *m*_1000_ denote the mass of the direct carbonation product after sintering and drying at 500 °C and 1000 °C, g. Here, 56 and 44 represent the molecular weights of CaO and CO_2_, respectively.

### 2.3. Material Characterization Methods

**Figure 1:**
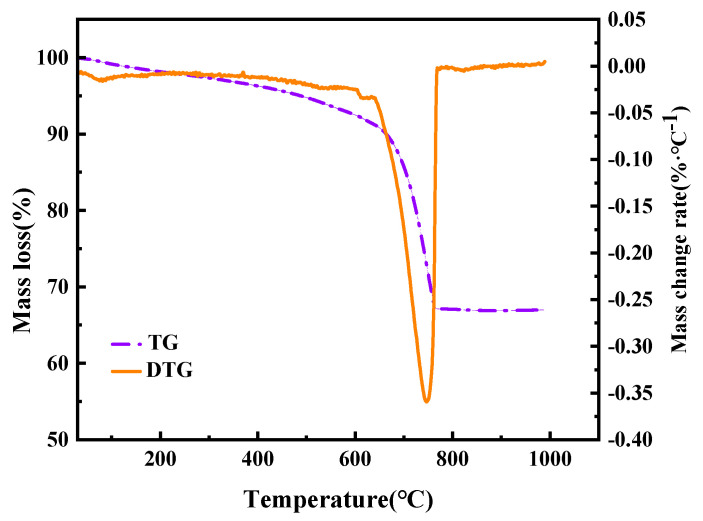
The thermogravimetric curves of indirect CO_2_ sequestration products at 60 °C.

To determine the elemental composition of the MS samples, quantitative analysis was performed on the preprocessed MS powder using an X-ray fluorescence spectrometer (Model: ZSX100e) equipped with a silicon drift detector (SDD). To identify the phase composition of the MS samples, XRD analysis was conducted on the preprocessed MS powder. The diffractometer (Model: Bruker D8) was coupled with an X’celerator detector using a copper (Cu) Kα X-ray source. The scanning range was 10° to 90°, with a scanning speed of 3.5 °/min. X-ray diffraction patterns were analyzed using JADE software (v.6.5). A field emission scanning electron microscope (SEM) model Quanta250FEG, FEI, Hillsboro, OR, USA was employed to analyze the microstructure of the MS samples. The samples were sputter-coated with a thin layer of gold (thickness: ~10 nm) to improve conductivity, and then observed under an acceleration voltage of 15 kV. TGA was performed on the MS samples subjected to indirect and direct carbonation using a thermogravimetric analyzer (Model: Q600) with a maximum measurement range of 1350 °C, a sensitivity of 0.1 ug. Mass loss was continuously recorded from 25 °C to 1000 °C at a heating rate of 5 °C/min. The thermogravimetric curves of the direct carbon sequestration products in this study are shown in Figure 1.

### 2.4. Life Cycle Assessment Methodology

Life cycle impact assessment (LCA) was performed with SimaPro 10.1.0.4 software, adopting the TRACI 2.2 method for impact characterization. This method quantifies potential environmental impacts across multiple categories, including global warming potential (GWP), acidification potential (AP), eutrophication potential (EP), ozone depletion potential (ODP), by applying region-specific characterization factors. This method covers the impact categories closely related to carbon sequestration and industrial waste utilization, and is suitable for both scientific research and industrial scenarios.

### 2.5. Visualization of Research Approach

**Figure 2:**
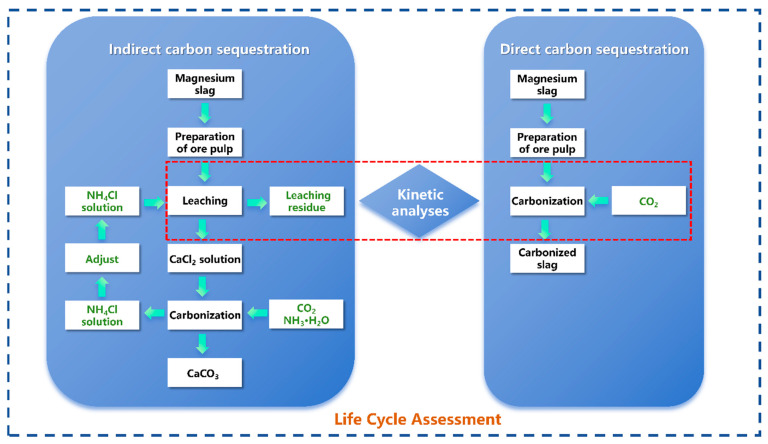
Research framework diagram.

This study established a systematic research framework comprising “dual-path process comparison, kinetic mechanism analysis, and full life cycle assessment.” First, experiments on direct carbonation and indirect carbonation were conducted using MS as raw material. The direct method involved carbonation by introducing CO_2_ gas directly into the MS slurry, whereas the indirect method utilized an ammonium chloride solution to leach calcium ions, followed by carbonation through the injection of CO_2_ gas. Second, to identify rate-limiting steps and mechanisms, this study conducted in-depth kinetic analyses of both pathways, calculating and evaluating kinetic equations and rate-influencing factors for each carbonation method. Finally, the Life Cycle Assessment (LCA) methodology was applied to quantify key indicators, such as Global Warming Potential (GWP), under both carbonation pathways, enabling a comprehensive environmental comparison and optimization. The research framework is illustrated in Figure 2.

## 3. Results and Discussion

### 3.1. Magnesium Slag Analysis

**Figure 3:**
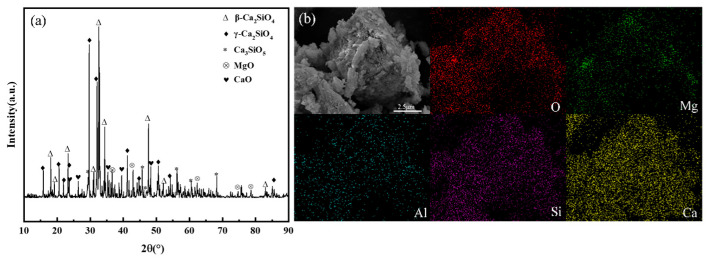
X-ray diffraction pattern and SEM-EDS image of MS sample (**a**) X-ray (**b**) SEM-EDS.

Table 1 indicates that the slag primarily consists of CaO, SiO_2_, and MgO, with relatively low Fe_2_O_3_ and Al_2_O_3_ content. Calcium, as the primary reactive element in mineral carbonation, its content level serves as an intuitive indicator to evaluate the carbonation potential of a mineral. With a CaO content as high as 56.66%, the MS is rich in calcium-bearing minerals, which makes it an ideal raw material for mineral carbonation-based CO_2_ sequestration and endows it with high utilization value. Phase analysis of the MS was conducted using X-ray diffraction (XRD), results are shown in Figure 3a. In MS, calcium exists predominantly in the form of crystalline mineral phases, including β-dicalcium silicate (β-Ca_2_SiO_4_), γ-dicalcium silicate (γ-Ca_2_SiO_4_), calcium sulfate pentoxide (Ca_3_SO_5_), along with magnesium oxide (MgO) and calcium oxide (CaO) as associated components. SEM-EDS analysis was performed on the MS samples to characterize their microtopography and elemental composition. The analytical results are presented in Figure 3b. These results indicate that, the particle size of the MS is non-uniform and has an irregular granular morphology, which is consistent with the result of sieving analysis (less than 74 μm). The main elements present were Ca, Si, O, and Mg.

### 3.2. Dynamics Research

**Figure 4:**
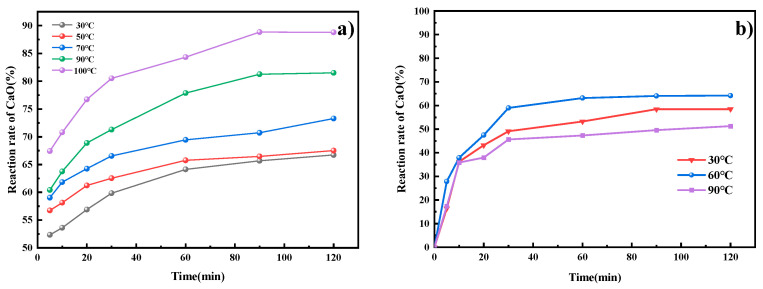
The effect of temperature on indirect and direct CO_2_ sequestration processes. (**a**) ICDS; (**b**) DCDS.

Temperature is a key factor influencing the choice of indirect and direct carbonation processes. The experimental temperature range of 30–100 °C encompasses both typical industrial leaching operating temperatures and the solubility range of CO_2_ in water. In the ICDS leaching process, Figure 4a shows that temperature significantly affects the reaction rate of CaO. As the temperature increases, the reaction rate of CaO rises. Within the same reaction time, higher temperatures yield a greater reaction rate of CaO. During the process of DCDS, Figure 4b reveals a stepwise effect of temperature on the reaction rate of CaO. The results indicate that the reaction rate of CaO in the DCDS process does not increase linearly with rising temperature but instead follows an initial increase followed by a decrease. Within the 30 °C to 60 °C temperature range, the reaction rate of CaO increases with temperature, indicating that the reaction rate accelerates with rising temperature, consistent with the Arrhenius equation. Reaction rate of CaO peaks at 60 °C. When temperatures exceed 60 °C (i.e., upon further heating to 90 °C), the reaction rate of CaO actually declines. This occurs because further temperature increases significantly reduce CO_2_ solubility, counteracting the acceleration effect of temperature on reaction rate.

To gain an in-depth understanding of the reaction pathways of the two carbon dioxide sequestration processes, reaction kinetic modeling was performed. In most solid–liquid reactions, the heterogeneous reaction kinetics of various ores can be described by the classical shrinking core model [17,18,19]. This model states that the reaction rate of the leaching process is mainly controlled by chemical reaction, intraparticle diffusion through the product layer, and mixed control. Furthermore, the reaction process is typically dominated by one of its steps. The primary reaction-controlling processes may include the following three types.

If the solid–liquid reaction process is controlled by chemical reactions, the reaction kinetic equation can be simplified to

(3) $$1-(1-X{)}^{\frac{1}{3}}=k_{1}t$$

If intra-product-layer diffusion limits the leaching process, the reaction kinetics equation can be simplified to

(4) $$1-\frac{2X}{3}-{\left(1-X\right)}^{\frac{2}{3}}=k_{2}t$$

If the reaction process is controlled by a combination of interfacial chemical reactions and diffusion within the product layer, the reaction kinetics equation can be simplified to

(5) $$\frac{1}{3\ln\left(1-X\right)}+{\left(1-X\right)}^{-\frac{1}{3}}=k_{3}t$$

In this equation, X represents the CO_2_ sequestration capacity (%); *k*_i_ (i = 1,2,3) denotes the kinetic constant of the leaching reaction control model; and *t* signifies the leaching time (minutes).

**Figure 5:**
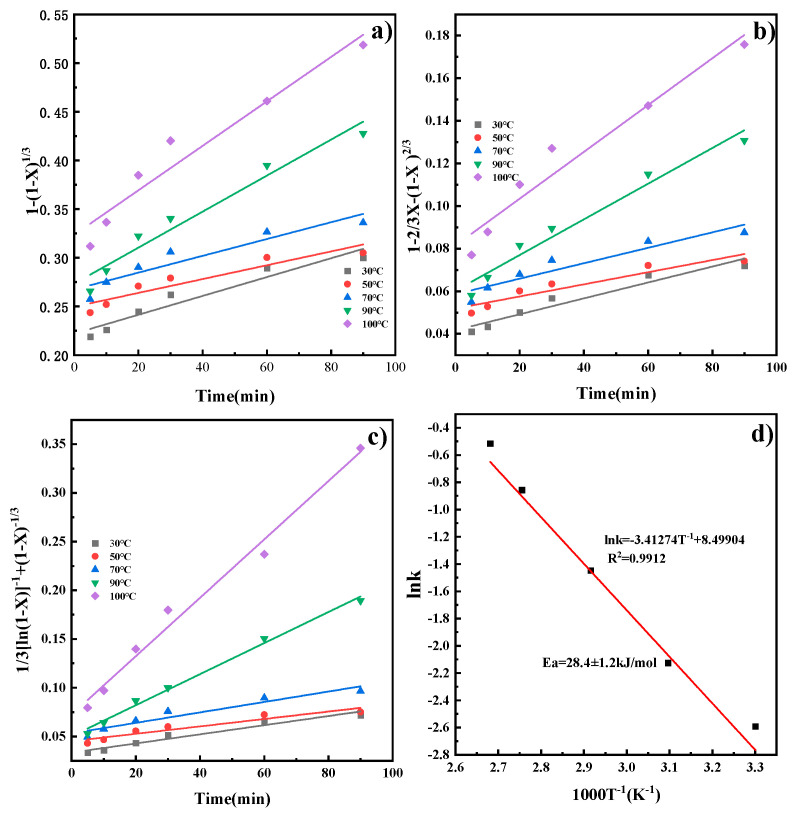
Kinetic model of ICDS reaction process and Arrhenius diagram (**a**) chemical reaction; (**b**) internal diffusion reaction; (**c**) chemical reaction and diffusion mixed reaction; (**d**) Arrhenius plot.

**Figure 6:**
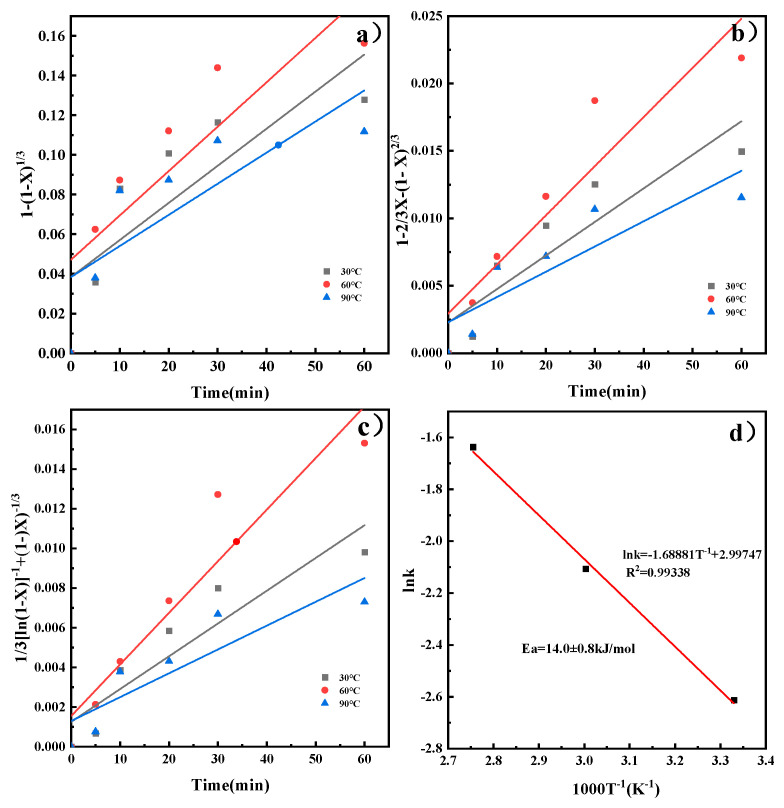
Kinetic model of DCDS reaction process and Arrhenius diagram, (**a**) chemical reaction; (**b**) internal diffusion reaction; (**c**) chemical reaction and diffusion mixed reaction; (**d**) Arrhenius plot.

By substituting the *X* at different temperatures into Equations (3)–(5), kinetic models for the reaction rate of CaO in the ICDS and the DCDS at various temperatures were obtained. These models are shown in Figure 5a–c, The apparent rate constants *k* and correlation coefficients R^2^ at different temperatures obtained from the figure are shown in Table 2 and Table 3. It can be concluded that the correlation coefficients in Figure 5c and Figure 6c are larger, and their fitting effects are optimal. Therefore, it can be determined that both CO_2_ sequestration methods are more consistent with the hybrid model.

The reaction rate constants were calculated from Figure 4a,b and Figure 5a–c were used to determine the activation energy according to the Arrhenius equation [20,21,22]:

(6) $$lnk=lnA\cdot (-\frac{E_{a}X}{RT})$$

Here, *k* is the reaction rate constant, *A* is the frequency factor (a pre-exponential factor in the Arrhenius equation), *E_a_* is the apparent activation energy (in kilojoules per mole), *R* is the gas constant (typically 8.314 J/(mol·K)), and *T* is the absolute temperature (in Kelvin).

Substituting the leaching reaction rate constant k at each reaction temperature *T* into Equation (6) for linear fitting yields the results shown in Figure 5d and Figure 6d. The plots reveal a linear relationship between *lnk* and 1000/T. Calculating the slope of the line yields apparent activation energies of 28.3 ± 1.2 kJ/mol and 14.0 ± 0.8 kJ/mol, respectively. The activation energy was calculated with a standard deviation of ±1.2 kJ/mol (ICDS) and ±0.8 kJ/mol (DCDS) using linear fitting of the Arrhenius equation, which reflects the reliability of the kinetic data. Activation energies for processes controlled by external diffusion or chemical reactions typically exceed 42 kJ/mol. Chemical reaction- and diffusion-mixed processes have activation energies from 12 to 42 kJ/mol. Processes controlled by internal diffusion show lower activation energies, generally between 4 and 12 kJ/mol [23]. This indicates that the calcium leaching process in indirect carbonation is under the combined control of chemical reaction and diffusion. The direct carbonation process is also governed by the same combined control mechanism, which is consistent with the results obtained from the previous analysis.

### 3.3. Study on Reaction Mechanism

**Figure 7:**
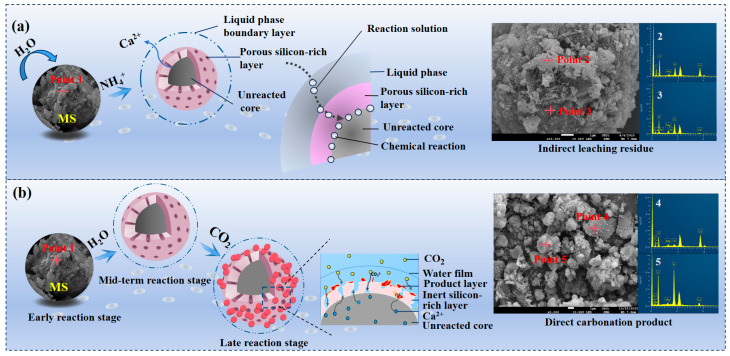
Carbonation reaction core contraction model (**a**) ICDS; (**b**) DCDS.

In the ICDS leaching process, the dynamic process of calcium leaching is illustrated in Figure 7a. During the initial stage, ion exchange occurs immediately upon contact between the ammonium chloride solution and MS. Calcium ions detach from the MS lattice and enter the solution, causing the calcium concentration to rise rapidly. The reaction is concentrated on the surface of the MS. During the intermediate stage, calcium ion depletion on the slag surface reduces leaching agent contact probability. Concurrently, partial calcium ion side reactions or re-adsorption occur, slowing the growth of the calcium concentration. In the late stage, calcium concentration stabilizes as the reaction approaches equilibrium, with nearly all leachable calcium ions entering the solution. During the reaction, a silicon-rich solid layer forms on the magnesia slag surface, impeding ion diffusion.

The DCDS process is based on the coupling of “two-step reactions + two-layer diffusion,” as illustrated in Figure 7b. During the initial reaction phase, the three-phase interface forms and ion dissolution initiates. After mixing MS with water to form a slurry and introducing CO_2_, the reaction commences at the “MS surface-water phase-CO_2_ bubble” three-phase interface. CO_2_ dissolves and ionizes (forming H_2_CO_3_ and H^+^, (HCO_3_)^−^, (CO_3_)^2−^), lowering the aqueous phase pH. The porous structure of the magnesia slag surface provides pathways for contact. CaO hydrate to form Ca(OH)_2_ and then dissolves into Ca^2+^. Directional dissolving of Ca^2+^ forms a silicate-rich layer. Increased (CO_3_)^2−^ concentration in the aqueous phase creates a mobile boundary between the silicate-rich layer and unreacted MS. H^+^ reacts with calcium silicates (e.g., Ca_2_SiO_4_) in the MS, causing Ca^2+^ to diffuse out of the lattice into the aqueous phase; inert SiO_2_ accumulates, forming a nanoscale “inert silica-rich layer” that gradually thickens and densifies, initially impeding ion transport. Calcium carbonate precipitates, and the product layer grows. Ca^2+^ diffuses to the “silicon-rich layer-aqueous phase” interface, reacting with (CO_3_)^2−^ to form a “product layer-silicon-rich layer” moving boundary.

During the mid-to-late reaction stages, diffusion resistance is coupled and controlled by both layers. The silicate-rich layer has extended pores and ion adsorption. The dense product layer is dominated by lattice diffusion with low coefficients. Together, they form a superimposed resistance that makes displacement reaction kinetics the rate-controlling factor. When ion transport rates fall below a threshold, the reaction slows. Ultimately, unreacted MS becomes encapsulated by both layers, and the reaction stops. The CO_2_ carbonation rate depends on the equilibrium between the thicknesses, porosity, and diffusion coefficients of the layers.

Table 4 presents the elemental distributions of raw MS, indirect leaching residue, and direct carbonation residue.

The EDS data intuitively reflect the elemental migration during ICDS and DCDS, consistent with the reaction mechanisms. Raw MS (Point 1) mainly contains O (67.7 at%), Ca (21.7 at%), Si (8.0 at%), and minor Mg (2.4 at%) without C, with Ca-containing phases as active components. After ICDS leaching (Points 2, 3), Ca content drops to 6.95~14.76 at %, Si rises to 21.86~26.65 at % (silicon-rich layer formation), and no C is detected. Following DCDS treatment (points 4 and 5), the presence of C confirms CO_2_ sequestration. Meanwhile, Ca and Si exhibit irregular, opposite distributions, indicating that the carbonation slag consists of CaCO_3_ products and unreacted SiO_2_.

### 3.4. Comparison of CO_2_ Sequestration Capacity

To more intuitively compare the CO_2_ sequestration efficiency of the two methods, the CO_2_ sequestration capacity is defined as the mass of CO_2_ absorbed by 100 g of MS, calculated using Formula (7).

(7) $$\beta =100w_{0}X\times (\frac{44}{56})$$

**Figure 8:**
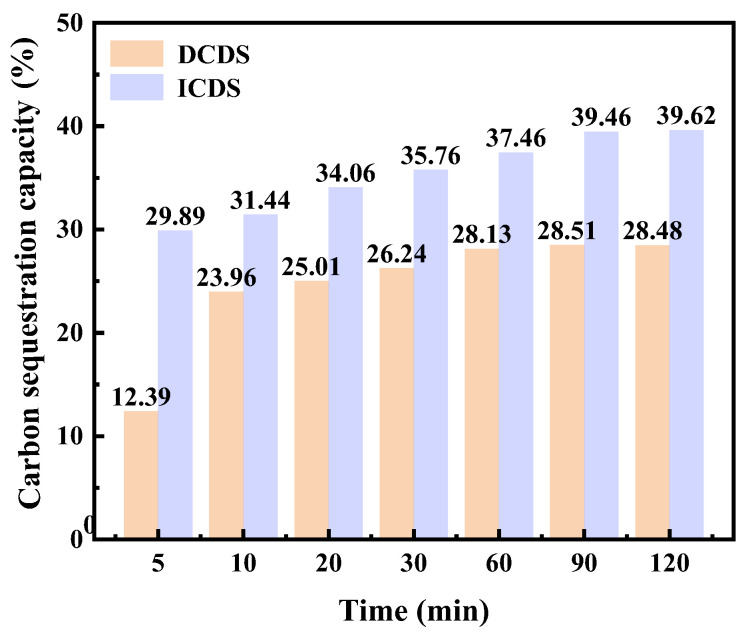
Comparative Analysis of CO_2_ sequestration capacity of DCDS and ICDS for MS.

β: CO_2_ sequestration capacity of 100 g MS, g/100 g. Under the optimal process conditions of the two carbon sequestration pathways (100 °C for the indirect pathway and 60 °C for the direct pathway), the experimental results are shown in Figure 8. The carbonation level of ICDS continuously increased throughout the entire period and then tended to stabilize. It steadily rose from 29.89% at 5 min to 39.46% at 90 min, and then increased further to 39.62% at 120 min, with almost no significant change, indicating obvious signs of saturation in the ICDS reaction. In contrast, the DCDS rate increased rapidly in the early stage and then stabilized over time. A significant growth was observed within the initial 60 min, increasing from 12.39% at 5 min to 28.13% at 60 min. After 60 min, the growth slowed down and stabilized at approximately 28% at 90 min (28.51%) and 120 min (28.48%), demonstrating that the DCDS reaction gradually reached saturation after 60 min.

Furthermore, the initial carbonation level of ICDS (29.89%) was higher than that of DCDS (12.39%), and it maintained a growth advantage throughout the entire process. This indicates that the ICDS capacity of MS is significantly greater than its DCDS capacity.

### 3.5. Life Cycle Assessment

**Figure 9:**
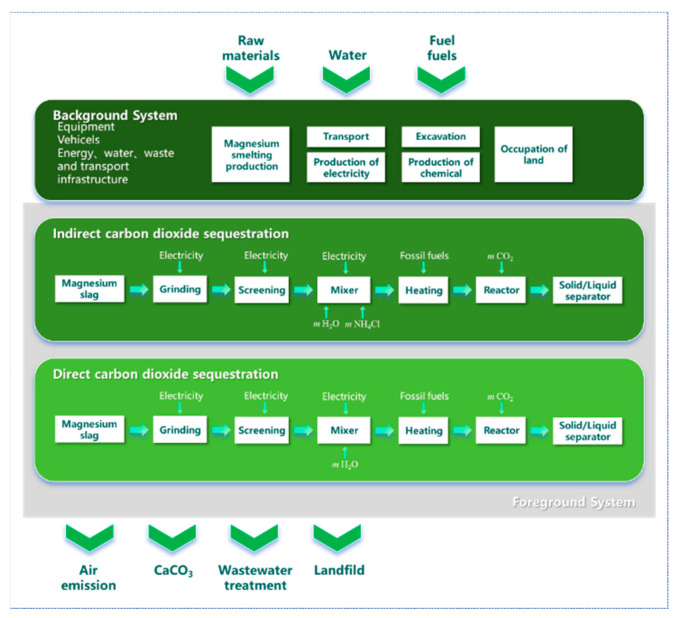
Life cycle assessment (LCA) boundaries.

TRACI 2.2 (Tool for the Reduction and Assessment of Chemical and other Environmental Impacts) is a life cycle impact assessment (LCA) methodology developed by the U.S. Environmental Protection Agency (U.S. EPA, Washington, DC, USA). As a mid-point level assessment model, it converts resource consumption and pollutant emissions into indicator values for several environmental impacts by assigning characterization factors to inventory data. In this study, SimaPro 10.1.0.4 was used to establish two CO_2_ sequestration process routes (DCDS and ICDS) for MS, and a LCA was conducted using the TRACI 2.2 methodology [24,25,26,27]. The process considered in this study is detailed in Figure 9, including the inputs, outputs, and a clear demarcation between the background and foreground systems. The functional calculation unit was 1 ton of MS for the entire CO_2_ sequestration life cycle process.

**Figure 10:**
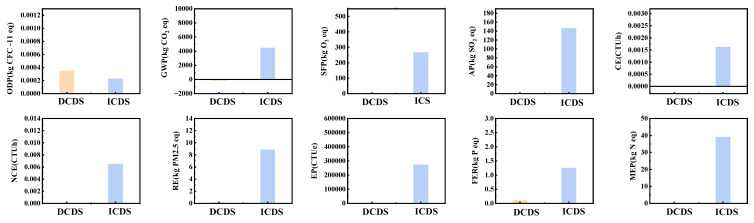
Comparison of characterization data from LCA between DCDS and ICDS processes.

This study conducts a comparative assessment of the environmental contributions of ICDS and DCDS across ten distinct dimensions. The objective is to systematically and comprehensively evaluate the actual performance of both technological routes, propose feasible recommendations for process improvement, and provide a valuable reference for optimizing project environmental performance. Specific numerical results are presented in Table 5 and Table 6, while a direct visual comparison of the data is illustrated in Figure 10.

The DCDS and ICDS exhibit stark differences in core environmental impact dimensions, with global warming potential (GWP) being the most pronounced. DCDS demonstrates significant CO_2_ sequestration benefits, while ICDS generates substantial CO_2_ emissions, creating a stark contrast. DCS has a climate change potential of −162.32 kg CO_2_ eq (negative values indicate CO_2_ sequestration), primarily stemming from the direct carbonization process itself, which alone achieves −284.85 kg CO_2_ eq of CO_2_ sequestration. This fully offsets emissions from tap water (84.59 kg CO_2_ eq), electricity (38.63 kg CO_2_ eq), and wastewater (14.81 kg CO_2_ eq). In contrast, the ICDS process exhibits a climate change potential of 4477.83 kg CO_2_ eq, with 88% of these emissions derived from ammonia usage (3949.68 kg CO_2_ eq). Ammonium chloride (1477.77 kg CO_2_ eq) and electricity (47.89 kg CO_2_ eq) further contribute to the emission burden. The difference in CO_2_ emissions between these two components reaches 4640 kg CO_2_ eq, equivalent to the emissions from burning 2 tons of standard coal.

Regarding acidification impacts, the acidification potential (AP) of DCS is only 0.53 kg SO_2_ eq, representing an extremely low environmental burden. In contrast, ICDS exhibits an AP of 146.48 kg CO_2_ eq, nearly 276 times that of DCDS. Approximately 75% of this acidification contribution stems from the ICDS process itself (110.52 kg SO_2_ eq), with acidic reagents used in the indirect carbonation process being the primary cause. In freshwater eutrophication potential (FEP), ICDS also significantly exceeds DCDS. Its potential of 1.26 kg P eq is 10.5 times that of DCDS (0.12 kg P eq). Ammonia usage (1.17 kg P eq, accounting for 93%) is the primary driver of aquatic eutrophication. The difference in ecotoxicity is even more striking. ICDS’s ecotoxicity potential (EP, 273,802.2 CTUe) is 146 times that of DCDS (1869.11 CTUe). Ammonia’s high ecotoxicity profile (290,191.53 CTUe, accounting for 99% of the total) makes it a critical threat to ecosystems. In terms of smog formation potential (SFP), ICS’s potential of 267.32 kg O_3_ eq is 49 times that of DCDS (5.47 kg O_3_ eq). As a key precursor to photochemical smog, ammonia contributes 89% of the smog formation load (237.15 kg O_3_ eq). Overall, DCDS demonstrates significant advantages across all five core environmental impact dimensions, exhibiting particularly qualitative superiority in CO_2_ sequestration and acidification control. Conversely, ICDS’s extensive use of ammonia reagents results in multiple environmental impact indicators exceeding standards, incurring higher environmental costs.

Beyond core environmental impacts, DCDS and ICDS also exhibit distinct characteristics in other dimensions such as ozone depletion potential (ODP), human health, and marine eutrophication (ME), with particularly significant differences observed in certain indicators. Regarding ODP, both processes exhibit extremely low impacts. DCDS has an ODP of 0.00035 kg CFC-11 eq, while ICDS is 0.66 times that of DCDS at 0.00023 kg CFC-11 eq. In this dimension, the risk of stratospheric ozone depletion from both is negligible. In the human health impact dimension, the differences between the two processes are polarized. Regarding carcinogenicity (CE), DCDS exhibits a slight negative contribution (−5.17 × 10^−6^ CTUh). In contrast, ICS generates a significant positive contribution (0.0016 CTUh), with a negative impact intensity 309 times that of DCDS. Ammonia usage (contribution: 0.0016 CTUh) is the primary driver of ICDS’s dramatically elevated carcinogenic risk. For non-carcinogenic effects (NCE), ICDS’s potential (0.0065 CTUh) also far exceeds DCDS (5.98 × 10^−5^ CTUh), reaching 109 times higher. Here, ammonia’s high non-carcinogenic toxicity (contribution: 0.0072 CTUh) is the dominant factor. Regarding respiratory effects (RE), ICDS exhibits a PM_2.5_ equivalent impact potential of 8.87 kg PM_2.5_ eq, which is 59 times the value for DCDS (0.15 kg PM_2.5_ eq). The ICDS technology itself (contributing 3.92 kg PM_2.5_ eq) is the primary driver of this significant increase in respiratory hazards. The most pronounced disparity emerged in marine eutrophication (ME), where ICDS exhibited a nitrogen equivalent potential of 39.09 kg N eq, 782 times that of DCDS (0.05 kg N eq). ICDS emissions (contributing 34.90 kg N eq) became the primary driver of ME, while DCDS demonstrated extremely low environmental impact in this dimension. Overall, except for ODP, where both impacts are negligible, ICDS exhibits higher environmental risks than DCDS across dimensions, including human health (CE, NCE), respiratory effects, and marine eutrophication (MEP). The disparities in MEP and CE are particularly pronounced, with the core cause directly linked to the use of ammonia reagents in ICDS processes.

**Figure 11:**
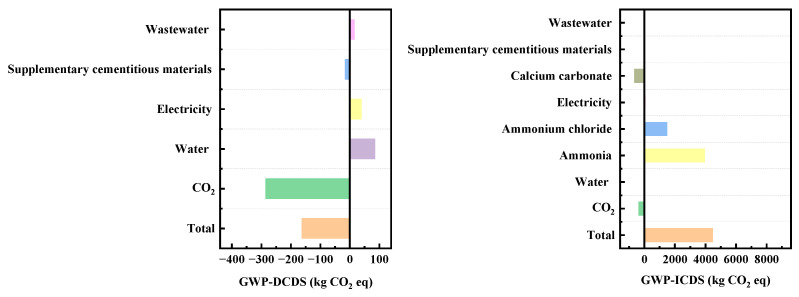
Analysis of GWP indicators for DCDS and ICDS processes.

As can be seen from Figure 11 on Greenhouse Gas Emissions (GWP), the emission contributions of specific materials such as ammonium chloride are clearly distinguishable in the GWP-ICDS chart. Indirect carbon emissions from ammonia-related processes represent a high-emission segment of the ICDS process. Optimizing the production processes of ammonia-based materials can achieve highly efficient emission reductions. Indirect embodied carbon emissions involve upstream or supporting links in the industrial chain, including electricity and supplementary cementitious materials (e.g., calcium carbonate). Compared with the control of direct embodied carbon emissions, emission reduction measures in the indirect embodied carbon dimension are more flexible and can deliver cross-link emission reduction benefits through industrial chain collaboration. The control of direct embodied carbon emissions mostly focuses on real-time optimization at the production end, with relatively weaker continuity of long-term benefits.

**Figure 12:**
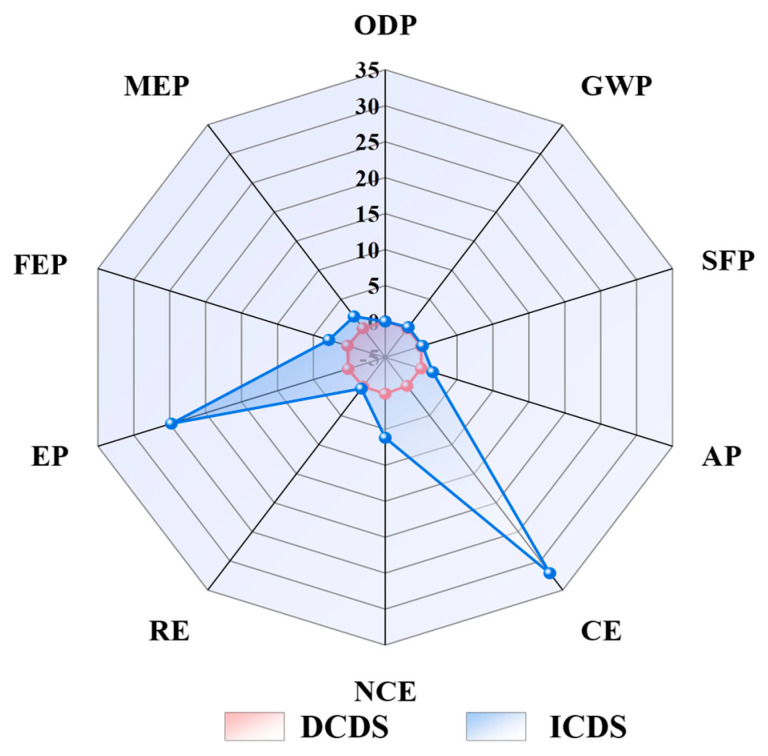
Comparison of normalized data between DCDS and ICDS. (GWP: global warming potential; AP: acidification potential; FEP: freshwater eutrophication potential; EP: ecotoxicity potential; SFP: smog formation potential; ODP: ozone depletion potential; MEP: marine eutrophication; CE: carcinogenicity; NCE: non-carcinogenic effects; RE: respiratory effects).

Although ICDS demonstrates superior efficiency compared to the DCDS, its associated environmental impacts are significantly more pronounced. Consequently, the selection of an appropriate CO_2_ sequestration technology in practical applications warrants careful deliberation. The analysis presented in the radar chart (Figure 12) indicates that the ICDS exhibits a substantially broader profile and more extensive implications across multiple critical environmental dimensions. In nine categories, including ecotoxicity, freshwater eutrophication, and marine eutrophication, the impact factors of ICDS significantly exceed those of DCDS. This signifies that the ICDS plays a dominant role in the progression of these environmental issues.

### 3.6. Comparison and Evaluation of DCDS and ICDS Processes

The DCDS process demonstrates comprehensive environmental sustainability advantages over the ICDS process, achieving a qualitative leap, particularly in climate change mitigation (CO_2_ sequestration) and acidification control. It represents a more viable and environmentally friendly option. However, relying on its core advantages of high raw material utilization rates, high product added value, and process flexibility, ICDS is also highly worthy of promotion in specific scenarios. Its “leaching-purification-carbonization” staged process overcomes mass-transfer limitations, achieving conversion rates of active components in MS that are over 30% higher than those in DCDS, making it particularly suitable for low-activity, high-impurity MS. Furthermore, ICDS enables the directional synthesis of high-value-added calcium carbonate with a purity exceeding 95%, whose economic value is 5–8 times higher than that of DCDS-derived products, thus conferring superior economic feasibility. The system also exhibits far greater flexibility than DCDS, as its operational parameters can be readily adjusted to accommodate raw materials with varying compositions.

To mitigate the environmental drawbacks of the ICDS process, three targeted optimization measures can be adopted to reduce its environmental footprint, as detailed below: First, anhydrous ammonia can be replaced with industrial waste lye, or over 90% of ammonia can be recycled; this approach can eliminate 88% of the primary CO_2_ emission sources. Second, the leaching pH can be adjusted and optimized to the range of 6.0–7.0, and dedicated wastewater treatment units can be incorporated to cut ecotoxicity by 95%. Third, conventional grid electricity can be substituted with renewable energy sources. After the implementation of these optimization strategies, the process’s global warming potential (GWP) can be reduced to below −50 kg CO_2_ eq, while ecotoxicity and other key environmental indicators can approach the levels of the direct carbonation system (DCDS). A core advantage of the ICDS process is its exceptional suitability for three specific scenarios: high-end calcium carbonate production, low-quality MS valorization, and co-disposal of industrial solid wastes. In summary, the optimized ICDS process achieves the goals of “advanced technology, economic viability, and environmental controllability”, rendering it a preferable alternative to DCDS for targeted applications.

## 4. Conclusions

This study addresses the key gaps in MS CO_2_ sequestration that involve unclear kinetic mechanisms and the lack of systematic life cycle assessment (LCA) for direct CO_2_ sequestration (DCDS) and indirect CO_2_ sequestration (ICDS), and it provides critical support for process optimization and engineering application through kinetic analysis and LCA.

First, the kinetic mechanisms of the two processes were clarified: ICDS follows a shrinking core model under mixed control (activation energy: 28.4 ± 1.2 kJ/mol) with a reaction rate of 89%, forming a silicon-rich inert layer. DCDS involves double-layer formation and pore blocking under mixed control (activation energy: 14.0 ± 0.8 kJ/mol) with a lower reaction rate of 63%. This reveals ICDS’s advantage in active component conversion and DCDS’s merit of low initiation energy consumption, filling the theoretical gap for MS CO_2_ sequestration industrialization. Second, LCA results differentiated the two processes: DCDS demonstrates excellent overall environmental sustainability, especially in CO_2_ sequestration and acidification control, making it an eco-friendly route for waste disposal and CO_2_ reduction; ICDS, despite severe environmental drawbacks (e.g., high CO_2_ emissions and ecotoxicity), boasts high raw material utilization and capacity for high-value-added calcium carbonate synthesis, suiting scenarios like low-grade MS resource utilization and high-end calcium carbonate production. Third, targeted optimizations (ammonia recycling, leaching pH regulation, renewable energy substitution) can reduce ICDS’s environmental load to near DCDS levels, achieving a balance of technical advancement, economic feasibility, and environmental controllability. Thus, DCDS is prioritized for large-scale eco-oriented projects, while optimized ICDS excels in high-value and low-quality MS disposal scenarios.

This study lays a theoretical foundation for MS carbonation process selection and promotion. Future work should focus on large-scale economic evaluation and long-term environmental monitoring to advance lab-to-industry translation, supporting the synergistic achievement of metallurgical solid waste valorization and dual-carbon goals.

## Figures and Tables

**Table 1:** Chemical composition analysis of MS.

Component	CaO	SiO_2_	MgO	Fe_2_O_3_	Al_2_O_3_	SO_3_	Others
Content, wt%	56.66	30.67	4.32	1.99	0.83	0.23	5.30

**Table 2:** Apparent rate constants and correlation coefficients of ICDS at different temperatures.

		1 − (1 − X)^1/3^		1 − 2/3X − (1 − X)^2/3^		1/3[ln(1 − x)]^−1^ + (1 − X)^−1/3^	
T[K]	1000/T	$k_{1}$	R^2^	$k_{2}$	R^2^	$k_{3}$	R^2^
303	3.300	0.00097	0.90782	0.00037	0.92184	0.00047	0.94194
323	3.096	0.00071	0.85300	0.00028	0.86567	0.00038	0.88717
343	2.916	0.00086	0.85376	0.00036	0.86949	0.00056	0.90094
363	2.755	0.00185	0.94550	0.00084	0.95991	0.00159	0.99154
373	2.681	0.00228	0.92586	0.00110	0.93743	0.00300	0.98252

**Table 3:** Apparent rate constants and correlation coefficients of DCDS at different temperatures.

		1 − (1 − X)^1/3^		1 − 2/3X − (1 − X)^2/3^		1/3[ln(1 − x)]^−1^ + (1 − X)^−1/3^	
T[K]	1000/T	$k_{1}$	R^2^	$k_{2}$	R^2^	$k_{3}$	R^2^
303	3.300	0.00187	0.60491	0.00025	0.78554	0.00017	0.81900
333	3.003	0.00224	0.66288	0.00036	0.84949	0.00026	0.87556
393	2.555	0.00157	0.53381	0.00019	0.70428	0.00012	0.83085

**Table 4:** EDS elemental composition of MS before and after CO_2_ sequestration.

At (%)	O	Si	Ca	Mg	C
Point 1	67.7	8.0	21.7	2.4	0
Point 2	58.42	26.65	6.95	6.71	0
Point 3	53.82	21.86	14.76	5.32	0
Point 4	40.58	29.70	8.75	1.04	19.92
Point 5	36.00	1.39	51.63	1.52	9.16

**Table 5:** LCA data by DCDS from MS.

Item	Unit	Total	DCS	Tap Water	Electricity, Low Voltage	Supplementary Cementitious Materials	Wastewater from a Vegetable Oil Refinery
GWP	kg CO_2_ eq	−162.32	−284.85	84.59	38.63	−15.5	14.81
AP	kg SO_2_ eq	0.53	0	0.41	0.17	−0.11	0.05
FEP	kg P eq	0.12	0	0.02	0.01	−0.0024	0.09
EP	CTUe	1869.11	0	1843.86	863.72	−1012.30	173.83
SFP	kg O_3_ eq	5.47	0	4.58	2.21	−2.13	0.81
ODP	kg CFC-11 eq	0.00035	0	0.00036	2.86 × 10^−7^	−2.00 × 10^−7^	1.13 × 10^−7^
CE	CTUh	−5.17 × 10^−6^	0	1.60 × 10^−5^	6.46 × 10^−6^	−3.02 × 10^−5^	2.60 × 10^−6^
NCE	CTUh	5.98 × 10^−5^	0	2.91 × 10^−5^	1.27 × 10^−5^	−8.99 × 10^−6^	2.71 × 10^−5^
RE	kg PM_2.5_ eq	0.15	0	0.11	0.05	−0.02	0.01
MEP	kg N eq	0.05	0	0.04	0.02	−0.02	0.01

**Table 6:** LCA data by ICDS from MS.

Item	Unit	Total	ICS	Tap Water	Electricity, Low Voltage	Supplementary Cementitious Materials	Wastewater from a Vegetable Oil Refinery
GWP	kg CO_2_ eq	4477.83	−375.77	22.56	47.89	−5.80	3.95
AP	kg SO_2_ eq	146.48	110.52	0.11	0.21	−0.04	0.01
FEP	kg P eq	1.26	0	0.0065	0.01	−0.00088	0.02
EP	CTUe	2.73 × 10^5^	0	491.70	1.07 × 10^3^	−378.89	46.35
SFP	kg O_3_ eq	267.32	0	1.22	2.74	−0.80	0.22
ODP	kg CFC-11 eq	0.00023	0	9.41 × 10^−5^	3.54 × 10^−7^	−7.49 × 10^−8^	3.01 × 10^−8^
CE	CTUh	0.0016	0	4.27 × 10^−6^	8.01 × 10^−6^	−1.13 × 10^−5^	6.93 × 10^−7^
NCE	CTUh	0.0065	0	7.76 × 10^−6^	1.57 × 10^−5^	−3.36 × 10^−6^	7.22 × 10^−6^
RE	kg PM_2.5_ eq	8.87	3.92	0.03	0.06	−0.0063	0.0033
MEP	kg N eq	39.09	34.90	0.01	0.02	−0.0077	0.0027
Item	Unit	Total	ICS	Ammonia, anhydrous, liquid	Ammonium chloride	Calcium carbonate, precipitated	
GWP	kg CO_2_ eq	4477.83	−375.77	3949.68	1477.77	−642.45	
AP	kg SO_2_ eq	146.48	110.52	35.30	6.11	−5.74	
FEP	kg P eq	1.26	0	1.17	0.23	−0.19	
EP	CTUe	2.73 × 10^5^	0	290 × 10^5^	2.96 × 10^5^	−4.72 × 10^4^	
SFP	kg O_3_ eq	267.32	0	237.15	65.36	−38.57	
ODP	kg CFC-11 eq	0.00023	0	9.51 × 10^−5^	5.66 × 10^−5^	−1.55 × 10^−5^	
CE	CTUh	0.0016	0	0.0016	0.00033	−0.00025	
NCE	CTUh	0.0065	0	0.0072	0.00044	−0.0012	
RE	kg PM_2.5_ eq	8.87	3.92	4.59	1.02	−0.75	
MEP	kg N eq	39.09	34.90	2.24	2.27	−0.36	

## Data Availability

The original contributions presented in this study are included in the article. Further inquiries can be directed to the corresponding authors.
